# Lateralization of the connections of the ovary to the celiac ganglia in juvenile rats

**DOI:** 10.1186/1477-7827-7-50

**Published:** 2009-05-21

**Authors:** Carolina Morán, Fabiola Zarate, José Luis Morán, Anabella Handal, Roberto Domínguez

**Affiliations:** 1 Department of Biology and Toxicology of Reproduction; Science Institute BUAP, Mexico; 2 Biology of Reproduction Research Unit; FES Zaragoza UNAM, Av. 14 sur 6301, San Manuel, Puebla, Pue. CP 72570, Mexico

## Abstract

During the development of the female rat, a maturing process of the factors that regulate the functioning of the ovaries takes place, resulting in different responses according to the age of the animal. Studies show that peripheral innervation is one relevant factor involved.

In the present study we analyzed the anatomical relationship between the neurons in the celiac-superior mesenteric ganglia (CSMG), and the right or left ovary in 24 or 28 days old female pre-pubertal rats. The participation of the superior ovarian nerve (SON) in the communication between the CSMG and the ovaries was analyzed in animals with unilateral section of the SON, previous to injecting true blue (TB) into the ovarian bursa. The animals were killed seven days after treatment. TB stained neurons were quantified at the superior mesenteric-celiac ganglia.

The number of labeled neurons in the CSMG of rats treated at 28 days of age was significantly higher than those treated on day 24. At age 24 days, injecting TB into the right ovary resulted in neuron stains on both sides of the celiac ganglia; whereas, injecting the left side the stains were exclusively ipsilateral. Such asymmetry was not observed when the rats were treated at age of 28 days.

In younger rats, sectioning the left SON resulted in significantly lower number of stained neurons in the left ganglia while sectioning the right SON did not modify the number of stained neurons. When sectioning of the SON was performed to 28 days old rats, no staining was observed.

Present results show that the number and connectivity of post-ganglionic neurons of the CSMG connected to the ovary of juvenile female rats change as the animal mature; that the SON plays a role in this communication process as puberty approaches; and that this maturing process is different for the right or the left ovary.

## Background

The innervation of the ovary involves sympathetic, parasympathetic and sensorial components of the autonomic nervous system and reaches the ovary through the ovarian plexus, the superior ovarian nerve (SON), and the vagus nerve [1-3]. The terminals of such nerves release a series of neurotransmitters to the interior of the gland; some of which have been considered regulators of steroidogenesis, early follicular development and ovulation [4-7].

The postganglionic perikarya of the ovarian fibers travel along the SON and the ovarian plexus nerve and come from the pre-vertebral ganglia of the celiac-superior mesenteric ganglia (CSMG) and other small ganglia located close to the origin of the ovarian and renal artery. The right and left parts of the CSMG are asymmetrical [1]. The vagus nerve has an important amount of synapses to the main CSMG [2].

Large neurons, small granular cells, described as small intensively fluorescent cells, (SIF); glial cells (Schwann and satellite cells); capilaries; mast cells; and fibroblasts can be observed in the CSMG. Fluorescence histochemical methods have localized catecholamines in sympathetic ganglia in ganglion cells bodies and SIF cells [8]. Several neuropeptides, including substance P (SP), vasointestinal peptide (VIP), gastrin and enkephalin [9] are localized in nerve fibers. This group of cells forms a barrier that surrounds the main neurons axonal cone [10,11].

In mammals, the CSMG is the main nervous information relay site between the gonads and the central nervous system; conversely, the link between the gonads and the central nervous system present modifications during the development of the ovaries [12,13]. The presence of neuropeptides, the topographic organization and maturing patterns of the neurons at the celiac ganglia of guinea pigs are set up at the end of the fetal period. After this period, neurons acquire their electro-physiological and morphological features [14].

The changes of the autonomic ganglia require neural interactions with its target organ. The nerve growth factor (NGF) and steroid hormones such as 17-β estradiol [15,16] control this regulation. Estradiol plays a critical role in regulating neuron survival and the way in which different neuron populations respond during the pre-natal and perinatal development [17,18]. According to Patrone *et al*. [19], during the early post-natal development estrogens increase the survival of dorsal root ganglia neurons.

In the spinal cord of the rat the mediolateral dendrites of preganglionic sympathetic neurons are detectable as early as the 15^th ^embryonic day [20]; and in the post- natal in the14^th ^day, the pattern of noradrenergic innervation was similar to that of the adult rat. Immunoreactive fibers were conspicuous in the intermediolateral and both thoracic and sacral levels. After day 14^th ^of age, a further overall increase in the density of innervations occurs [21]. In rats the ovarian innervation appears before birth and precedes the postnatal initiation of folliculogenesis, suggesting that the initial formation of follicles is, at least in part, facilitated by signals of neural origin [22]. The ovary of the rhesus monkey undergoes changes in nerve fibers density reaching the ovaries, increasing gradually since the postnatal stage, while during the pre-pubertal stage the density of nerve fibers increases at twice the rate [23]. D'Albora *et al*. [24] found changes in number, morphology and distribution of ovarian neurons in the Wistar rat, depending on the age of the animal.

Flores *et al*. [25] showed that in denervated pre-pubertal rats treated with guanethidine, the number of ova released during the first estrus was higher than in control rats. Guanethidine treatment to adult rats resulted in a significant reduction in the number of ova shed, suggesting that the ovarian noradrenergic innervation regulating ovulation plays different roles in pre-pubertal and adult rats. Chavez et al. [26,27] showed that ovulation processes are impacted when the SON is sectioned suggesting that the participation of the innervation on ovarian regulation varies during the estrous cycle and is asymmetric.

Previously we have shown that sensorial denervation through capsaicin treatment at birth, previous to folliculogenesis, results in a reduction of steroidogenesis and an increase in the number of 100–350 μm follicles. Most of these follicles were normal when the animals were studied on day 24 of age, and atretic when animals reached 28 days of age [28].

Similar differences on the effects of denervation between 24 and 28 days old rats, were observed in rats with unilateral or bilateral sectioning of the SON or vagus nerve [29-31], suggesting that innervation plays different roles on the regulation of ovarian development. It has been suggested that at age 28 days the system would be starting to prepare to reach the sexual mature stage [28].

To our knowledge, no quantitative morphological study of ovarian innervation has been conducted throughout the juvenile period of the rat, and thus, it is not clear whether the observed changes reflect metabolic or structural alterations. The aim of the present study was to determine whether structural remodeling of ovarian innervation in CSMG occurs during the pre-pubertal period and if the neural information amount between the ovaries and the CSMG is different between the left and right sides.

## Methods

The study was conducted with virgin adult female rats of the CIIZ-V strain, reproduced and maintained in the "Claude Bernard" Animal House in the Universidad Autónoma de Puebla. Animals were kept under controlled lighting conditions (lights on from 07:00 to 19:00 h), with free access to the mother until weaning (day 21) and to food (Purina S.A., Mexico) and tap water thereafter. The experiments were carried out in strict accordance with the Mexican Guide for Care and Use of Laboratory Animals at the National Academy of Science Animals and Treatment. Protocols were approved by the FES Zaragoza, UNAM. At birth (day 1) the animals were randomly allotted in groups of 5 females and 1 male to one of the experimental groups described below.

The injection of TB was performed following previously described procedures [32]. In brief, the animals were ether anesthetized between 10:00 am and 12:00 PM, and a unilateral incision was performed 1 cm below the last rib, affecting skin, muscle, and peritoneum. The left or right ovary was exposed and 3–5 μL of TB (Sigma, St. Louis, Missouri, USA) solution at 4%, diluted in distilled water, was injected into the ovarian bursa. To prevent the leakage of the tracer, the needle was kept in the bursa for 5 min after injection treatment. Subsequently, the ovary was carefully cleaned, dried, and returned to the abdominal cavity. The possibility that TB leaked into the abdominal cavity was assessed by exposing the cavity to a fluorescent light, and the animals with TB leakage were excluded from the experiment.

In other groups, the animals were treated as previously, but before the TB treatment into the bursa, the ipsilateral SON to the ovary to be treated was sectioned, following previously described methodologies [27].

Seven days after surgery the rats were anesthetized with sodium pentobarbital (40 mg/Kg) IP and intracardiac perfused with 250 mL of cold saline solution (0.9%), followed by the injection of 150 mL solution of 4% paraformaldehyde. After perfusion of the fixative solution, the pre-vertebral CSMG, ovaries, and uterus were dissected and kept in the fixative solution overnight (approx 18 h). The ganglia were cryoprotected successively in 10, 20, and 30% sucrose in phosphate buffer and were serially sectioned at twenty micrometer with the aid of a cryostat kept at -20°C. The sections were analyzed following the same procedure previously described [32]. In brief, eight to ten images of the right or left CSMG, from the animals injected with TB, were used to count the number of positively labeled cells. Positive TB cells are defined as cells in which fluorescence is present when the sections were exposed to UV light. Only principal neurons were labeled with TB and SIF neurons are not labeled with the tracer.

The pictures were obtained with a Digital Camera (Optronics 60300, USA) and analyzed with a KS-300 Imaging System 3.0 (Carl Zeiss Vision GmbH, Germany). The imaging system was programmed to generate binary regions, automatically count and integrate them.

### Statistical Analysis

The mean number of True Blue positive cells was analyzed by a multivariate analysis of variance (MANOVA), followed by Tukey's test. A p < 0.05 was assumed as significant.

## Results

**Figure 1:**
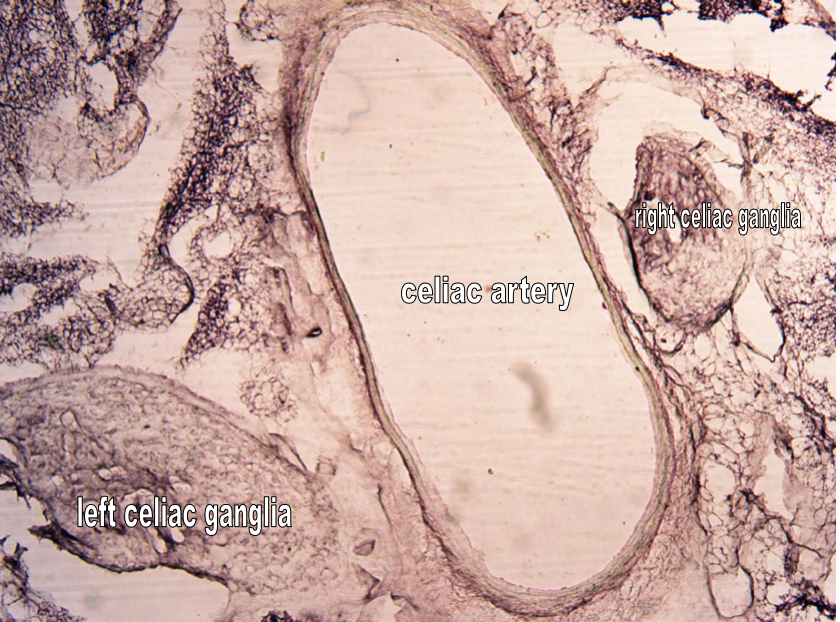
**The left and right CSMG**. 4×.

In figure 1 the left CSMG and right CSMG are showing.

**Figure 2:**
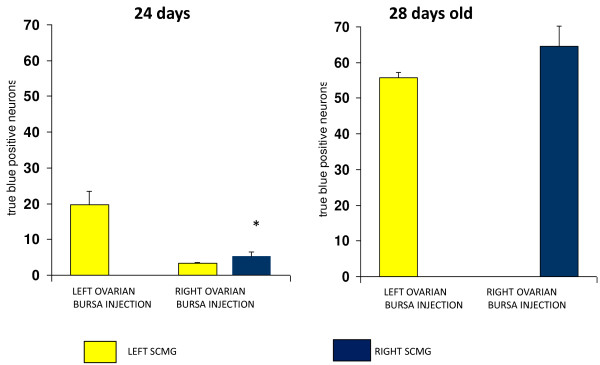
**Number of positive neurons to true blue in the celiac superior-mesenteric ganglia of the animals injected with the true blue in the left or right ovarian bursa at 24 or 28 days old, and sacrificed seven days later**. * p < 0.05 vs. contralateral CSMG.

Number of positive neurons to TB in the CSMG of the animals injected with the tracer in the left or right ovarian bursa (Figure 2).

**Figure 3:**
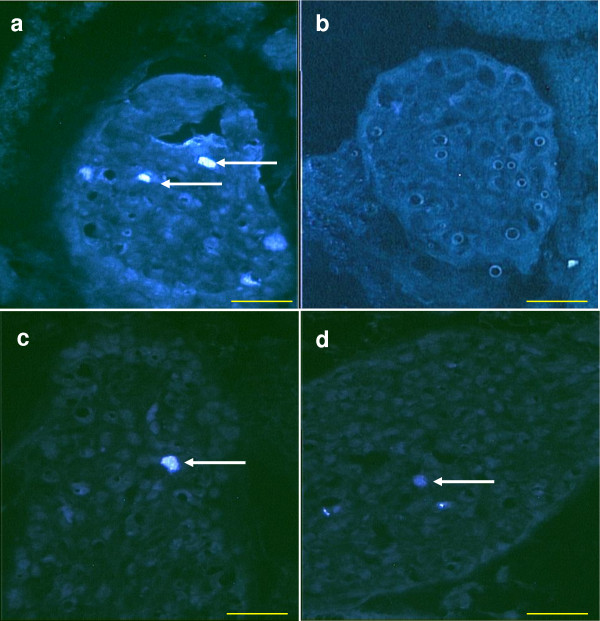
**True Blue labeled neurons (white arrows) in the CSMG 24 days old rats injected with TB in the left (a, b) or into the right ovary (c, d)**. Left CSMG a and c; Right CSMG b and d. Scale bar 100 μm.

24 days old. The injection of TB into the right ovarian bursa resulted in stained neurons in both, left and right CSMG. When the tracer was injected into the left ovarian bursa, stained neurons were observed only in the left CSMG (Figure 3), and the number of stained neurons in the left CSMG was significantly higher than when the tracer was injected into the right ovarian bursa.

**Figure 4:**
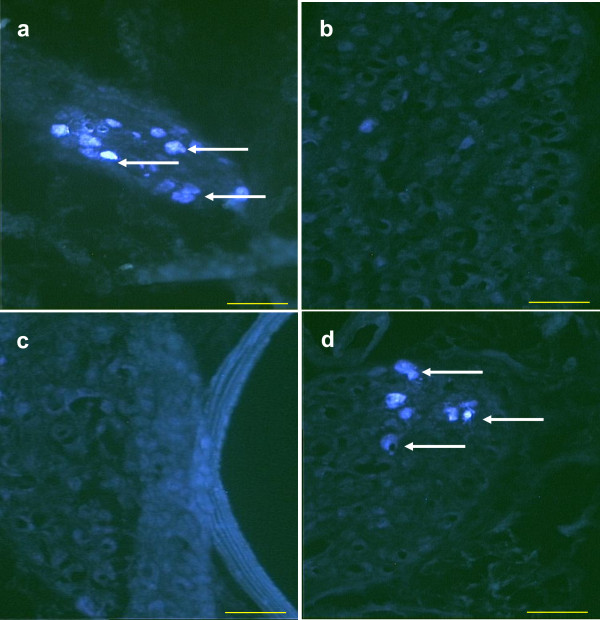
**True Blue labeled neurons (white arrows) in the CSMG 28 days old rats injected with TB in the left (a, b) or into the right ovary (b, c)**. Left CSMG a and c; Right CSMG b and d.

28 days old. The number of TB stained neurons was significantly higher than in rats injected at 24 days of age. Significant differences in the number of TB positive cells in the left and right CSMG were not observed. Unlike what was observed in animals treated at 24 days, injecting TB into the right or left ovary to 28 days old rats resulted exclusively in stains neurons of the ipsilateral CSMG than the treated ovary (Figure 4).

**Figure 5:**
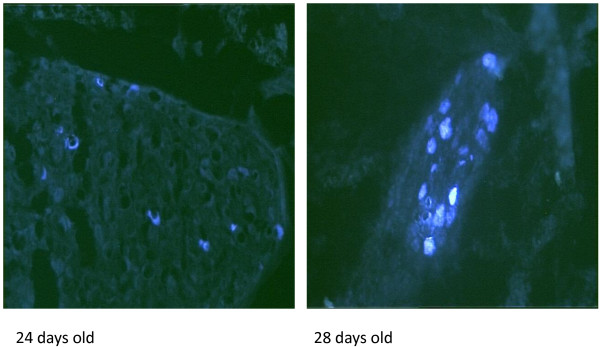
**Diferences in the intensitity of TB positive cells in the CSMG of 24 and 28 days old rats**.

In figure 5 we show the differences in the fluorescence intensity in the TB positive cells in the CSMG of 24 and 28 days old rats.

Number of TB positive neurons in the CSMG of animals with unilateral sectioning of the SON, injected with TB in the left or right ovarian bursa.

**Figure 6:**
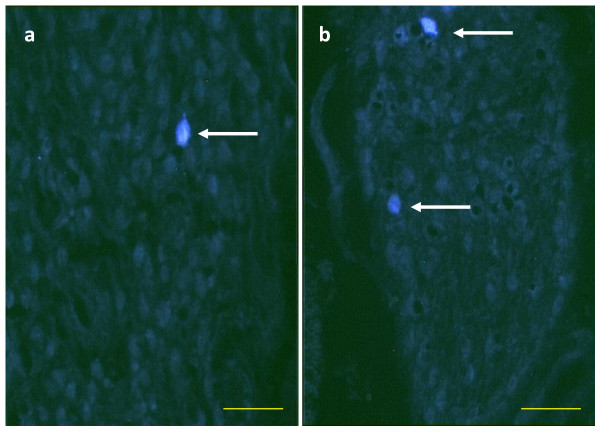
**True Bleu labeled neurons (white arrows) in the CSMG of 24 days old rats with section of the left SON injected with TB in the left ovary (a) or with section of the right SON injected with TB into the right ovary (b)**.

24 days old. When the left SON was sectioned, the number of TB stained neurons in the left CSMG diminishes significantly. This effect was not observed in the right CSMG when the right nerve was sectioned (Table 1, and Figure 6).

**Table 1:** Number of positive neurons in CSMG of the rats injected with TB in the 6 left or right ovarian bursa posterior to the section of the ipsilateral SON.

Group	Ovarian bursa injected	Control	After SON section
24 days old	Left	19.8 ± 3.6	7.7 ± 2.4 ♠
24 days old	Right	5.2 ± 1.3*	5.6 ± 1.1
28 days old	Left	55.8 ± 1.5	0
28 days old	Right	64.6 ± 15.7	0

* p < 0.05 vs. rats injected with TB in the left ovarian bursa

♠ p < 0.05 vs. control rats injected with TB in the left ovarian bursa

28 days old. When the SON was sectioned at this age, no stained neurons were observed in the ipsilateral CSMG, independently of the side in which the section-TB injection was applied (Table 1).

## Discussion

The results obtained in the present study show that in rats, during the juvenile and pre-pubertal period arrangements in the post-ganglionic neurons reaching the ovaries that arrive from the pre-vertebral ganglia, take place. The results also suggest that the SON plays an important role in the communication process between the CSMG and the ovaries as puberty approaches.

Nerve terminals are present in the rat's ovaries since very early stages of embryonic development, increasing in number and density until puberty onset; and many of them are catecholaminergic [13,22,33-35].

Present results support the idea of the presence of two celialic ganglions (left and right) and that the left celialic ganglion is larger than the right one [1]; and differ from Burden and coworkers [3,33] who described the presence of a single celialic ganglion. The difference if the number of celialic ganglions reported by Burden et al and those observed in this investigation may be explained by the different rat strains used.

In this study, differences in the number of TB stained CSMG neurons, between rats treated at the age 24 and 28 days, suggest that at these two development stages there are differences in the level of information transmitted between the pre-vertebral ganglia and the ovaries. This difference may be related to the maturation processes because the number of innervations observed in the CSMG in 28 days old animals is similar to those observed in adult cyclic rats. In a previous study rats injected with TB directly into the ovaries, where animals treated throughout the estrous cycle showed an increase in the number of stained neurons from diestrus 1 to proestrus, with a small drop in animals treated on estrus. These results were interpreted as suggesting that the number of active neurons in the CSMG varies during the estrous cycle. Such changes could be related to oscillating estradiol plasma levels and to changes in the neurons' ability to be stimulated by estrogens throughout the estrous cycle [32].

There is evidence of functional asymmetry between paired organs [36-38]. In cyclic rats the left ovary releases more ova than the right one [39,40]. Morphological asymmetry in the intensity of supraespinal innervation of the left and right ovary has been showed by Tóth *et al*. [41]. The neural connections between the left ovary and brain structures, such as the nucleus of the solitary tract, the dorsal nucleus of the vagus, the A5 noradrenergic cell group, the caudal raphe nuclei, the hypothalamic paraventricular nucleus, and the lateral hypothalamus, are more abundant than the number of neural connections between these cell groups and the right ovary [41].

Previously we have shown that the unilaterally sectioning the SON of pre-pubertal rats results in puberty onset delay and lower number of oocytes released by the denervated gonad [42,28]. The sequential injection of gonadotropins to these animals did not reestablish ovulation in the denervated ovary [30]. In the ovaries, asymmetric functions did not seem to depend on the gonadotropic stimuli, but on the extrinsic innervations received by each ovary [39].

In the CSMG of rats injected with TB into the right ovarian bursa at 24 days of age, the number of stained neurons in the left and right ganglia was lower than in animals injected with the tracer in the left ovary; a difference not observed in 28 days old animals. In the adult rat the highest number of stained cells was observed when the tracer was injected into the left ovary on proestrus. The number of stained cells was significantly different between the right and left CSMG of animals injected on proestrus, and bilateral staining in the CSMG was more pronounced when the tracer was injected into the left ovary. Such communication could partially explain the response differences to peripheral denervation observed in the right and left ovaries [30].

In 24 days old animal TB treatment in the right ovarian bursa resulted in a bilateral staining of neurons in the CSMG, while treatment in the left ovary resulted in staining of neurons on the ipsilateral side only. Such behavior supports the idea of a neural communication between the ovaries, partially explaining the difference in the response of the left and right ovaries to unilateral denervations [42,28,11].

The CSMG is the origin of the two catecholaminergic pathways innervating the ovary, the SON and the ovarian plexus nerve. The fact that in the 28 days old animal sectioning the SON eliminated the presence of TB stained neurons in the CSMG, we presume that the neuron subpopulation in the ganglia projecting its axons to the gonad through the SON has been developed.

Given that in 24 days old animals sectioning of the left SON results in a decrease of TB stained neurons in the CSMG, though not completely eliminating the presence of stained cells, it is proposed that during the first part of the juvenile period the neural connection between the left CSMG and the left ovary is carried through the nerve of the ovarian plexus and the SON. The neural connection between the right CSMG and the right ovary is carried only by the nerve of the ovarian plexus, because the section of the right SON did not modify the number of labeled cells.

Because the amount of tracer (TB and horseradish peroxidase) detectable in the cell body is correlated with functional activity of the neuron [43], and there is evidence that the capacity of the ovary to release noradrenaline increases when the rat reaches puberty [13], we presume that the activity of the CSMG cells related with the ovaries increases its activity when the animal reaches the puberty.

Present results support the hypothesis that the participation of the peripheral nerves on ovarian performance varies according with the age, and is asymmetric [28,42,44,45].

Taken together, present and previous results, suggest that in the rat: 1) The variations in response of both ovaries -along their development- are due to the rearrangement of the neurons and the fibers that convey information from the prevertebral ganglia to the gonads. 2) That the participation of the SON becomes more important for such communication as puberty approaches.

## Competing interests

The authors declare that they have no competing interests.

## Authors' contributions

CM and RD devised the study, participated in the discussion of results and in the planning of experiments; CM, and FZ, conducted the descriptive and quantitative histological studies; JLM and AH participated in the discussion of results. All authors read and approved the final manuscript.
